# Gut microbiota and autism spectrum disorders: a bidirectional Mendelian randomization study

**DOI:** 10.3389/fcimb.2023.1267721

**Published:** 2023-12-14

**Authors:** Zhi Li, Shuai Liu, Fang Liu, Nannan Dai, Rujia Liang, Shaoguang Lv, Lisha Bao

**Affiliations:** ^1^ Department of Pediatrics, Hebei Medical University, Shijiazhuang, Hebei, China; ^2^ Department of Pediatrics, Bethune International Peace Hospital, Shijiazhuang, Hebei, China; ^3^ Department of Cancer Epidemiology Division, University of Hawaii Cancer Center, Honolulu, HI, United States; ^4^ Department of Clinical Laboratory, The ECO-City Hospital of Tianjin Fifth Central Hospital, Tianjin, China

**Keywords:** gut microbiota, autism spectrum disorders, mendelian randomization, causal role, single nucleotide polymorphism

## Abstract

**Background:**

In recent years, observational studies have provided evidence supporting a potential association between autism spectrum disorder (ASD) and gut microbiota. However, the causal effect of gut microbiota on ASD remains unknown.

**Methods:**

We identified the summary statistics of 206 gut microbiota from the MiBioGen study, and ASD data were obtained from the latest Psychiatric Genomics Consortium Genome-Wide Association Study (GWAS). We then performed Mendelian randomization (MR) to determine a causal relationship between the gut microbiota and ASD using the inverse variance weighted (IVW) method, simple mode, MR-Egger, weighted median, and weighted model. Furthermore, we used Cochran’s Q test, MR-Egger intercept test, Mendelian Randomization Pleiotropy RESidual Sum and Outlier (MR-PRESSO), and leave-one-out analysis to identify heterogeneity and pleiotropy. Moreover, the Benjamin-Hochberg approach (FDR) was employed to assess the strength of the connection between exposure and outcome. We performed reverse MR analysis on the gut microbiota that were found to be causally associated with ASD in the forward MR analysis to examine the causal relationships. The enrichment analyses were used to analyze the biological function at last.

**Results:**

Based on the results of IVW results, genetically predicted *family Prevotellaceae* and *genus Turicibacter* had a possible positive association with ASD (IVW OR=1.14, 95% CI: 1.00-1.29, *P*=3.7×10^−2^), four gut microbiota with a potential protective effect on ASD: *genus Dorea* (OR=0.81, 95% CI: 0.69-0.96, *P*=1.4×10^−2^), *genus Ruminiclostridium5* (OR=0.81, 95% CI: 0.69-0.96, *P*=1.5×10^−2^), *genus Ruminococcus1* (OR=0.83, 95% CI: 0.70-0.98, *P*=2.8×10^−2^), and *genus Sutterella* (OR=0.82, 95% CI: 0.68-0.99, *P*=3.6×10^−2^). After FDR multiple-testing correction we further observed that there were two gut microbiota still have significant relationship with ASD: *family Prevotellaceae* (IVW OR=1.24; 95% CI: 1.09-1.40, *P*=9.2×10^-4^) was strongly positively correlated with ASD and *genus RuminococcaceaeUCG005* (IVW OR=0.78, 95% CI: 0.67-0.89, *P*=6.9×10^−4^) was strongly negatively correlated with ASD. The sensitivity analysis excluded the influence of heterogeneity and horizontal pleiotropy.

**Conclusion:**

Our findings reveal a causal association between several gut microbiomes and ASD. These results deepen our comprehension of the role of gut microbiota in ASD’s pathology, providing the foothold for novel ideas and theoretical frameworks to prevent and treat this patient population in the future.

## 1 Introduction

According to the Diagnostic and Statistical Manual of Mental Disorders (5th edition) criteria, autism spectrum disorder (ASD) is defined as a group of neurodevelopmental disorders characterized by deficits in social communication, social interaction, restricted or repetitive behavior, and interests (American Psychiatric Association, 2013). The global prevalence of ASD has progressively climbed within the past years, with the World Health Organization projecting that ASD will affect one in every 36 people worldwide by 2022 (Maenner et al., 2023). Caring for a child with ASD can be extremely challenging for caregivers, resulting in significant psychological, emotional, and financial hardships. Additionally, caregivers often lack adequate social support. According to a survey conducted in Saudi Arabia in 2022, only 31% of children with ASD had access to ASD facilities in their vicinity, while 72% had no local private schools catering to ASD (Khan et al., 2020). It is now understood that caregivers of children with ASD face a greater burden on their family resources and overall functioning than those caring for children with other developmental disabilities (Rogge and Janssen, 2019). Therefore, ASD has become a major public health concern in many countries (Yousef et al., 2021; Alenezi et al., 2022; De Jonge et al., 2023). The exact causes and factors that increase the risk of ASD remain poorly understood. Still, various potential contributors have been recognized, encompassing prenatal and perinatal periods, as well as exposure to specific environmental factors (Weir et al., 2021).

It is well-established that people with ASD suffer from various developmental abnormalities and experience additional symptoms such as gastrointestinal issues, seizures, anxiety, sleep disturbances, and immune system dysfunction (Kohane et al., 2012; Ballini et al., 2019; Hossain et al., 2020). Besides, children with ASD may be prone to experiencing gastrointestinal problems like diarrhea, constipation, and abdominal pain (Ferguson et al., 2019). There is an increasing consensus suggesting an association between gastrointestinal microbiota and ASD. In this respect, significant heterogeneity has been documented in the composition of the intestinal flora of ASD patients (Ferguson et al., 2019). Numerous prior investigations have indicated the potential role of the gut microbiome in ASD. The underlying mechanisms involve intricate and bidirectional interactions between the gut microbiota and the brain, which occur along the microbiota-gut-brain axis (Yu et al., 2021). Due to the predominantly observational nature of existing studies and the potential influence of confounding variables, the current evidence only establishes an association between gut microbiota and ASD without establishing a cause-and-effect relationship. Besides, the effects of this association on the incidence of ASD and its specific physiological mechanisms are still unclear, and whether this association is violated by reverse causality remains undetermined.

Mendelian randomization (MR) analysis is a novel genetic epidemiological study design that harnesses genetic variation to address causal questions related to the influence of modifiable exposures on different outcomes (Davey Smith et al., 2020; Sanderson et al., 2022). The MR approach draws on Mendel’s first and second laws of genetic inheritance: the law of segregation and the law of independent assortment avoids bias from unobserved confounding of exposure and outcome and can infer causal effects in the presence of unobserved confounding (Davey Smith and Ebrahim et al., 2003). Genome-wide association studies (GWAS) provide vast datasets with numerous single nucleotide polymorphisms (SNPs) and large sample sizes. MR, which employs one or more genetic polymorphisms as the exposure variable of interest, capitalizes on the Mendelian laws of inheritance, making GWAS-based MR an appealing approach to assess causality (Thanassoulis and O’Donnell, 2009; O’Donnell and Sabatine, 2018; Davey Smith et al., 2020; Sanderson et al., 2022). Kantan originally proposed the MR causation hypothesis, he stating that the APOE2 gene variant occurs randomly at birth, that the variation is not affected by confounding factors, and is associated with lower cholesterol levels. If there is a clear causal relationship between low cholesterol levels (exposure factors) and cancer (outcome), then people with APOE2 variants (instrumental variables) will be more susceptible to cancer. The link between APOE2 and cancer may provide indirect evidence of whether there is a causal relationship between serum cholesterol and cancer (Katan, 1986). A large-scale GWAS investigating the impact of host genetic loci on the gut microbiota has facilitated MR studies of gut flora (Kurilshikov et al., 2021). Moreover, the MR analysis is commonly described as a “naturally occurring randomized controlled trial” due to the random assortment of genetic variation during meiosis (following Mendel’s first and second laws), thus it has recently become popular in investigating the potential causal effects of the gut microbiota on different disease (Li et al., 2023; Luo et al., 2023; Sha et al., 2023; Yuan et al., 2023). This study utilized the latest GWAS data on ASD and gut microbiota to ascertain the causal correlation between gut microbiota and ASD. The reverse MR analysis was also conducted to bolster the findings’ dependability. Finally, we performed gene ontology (GO) analysis based on lead SNPs to explore the biological role of gut microbiota on ASD. We have identified specific gut microbiota in increasing or reducing the risk of ASD. Some of them have not been incorporated into the observational studies of ASD. The GO study provided more insights into the underlying physiology mechanism. Our study provides new directions and original insights into future studies on ASD.

## 2 Methods

### 2.1 Study design

**Figure 1:**
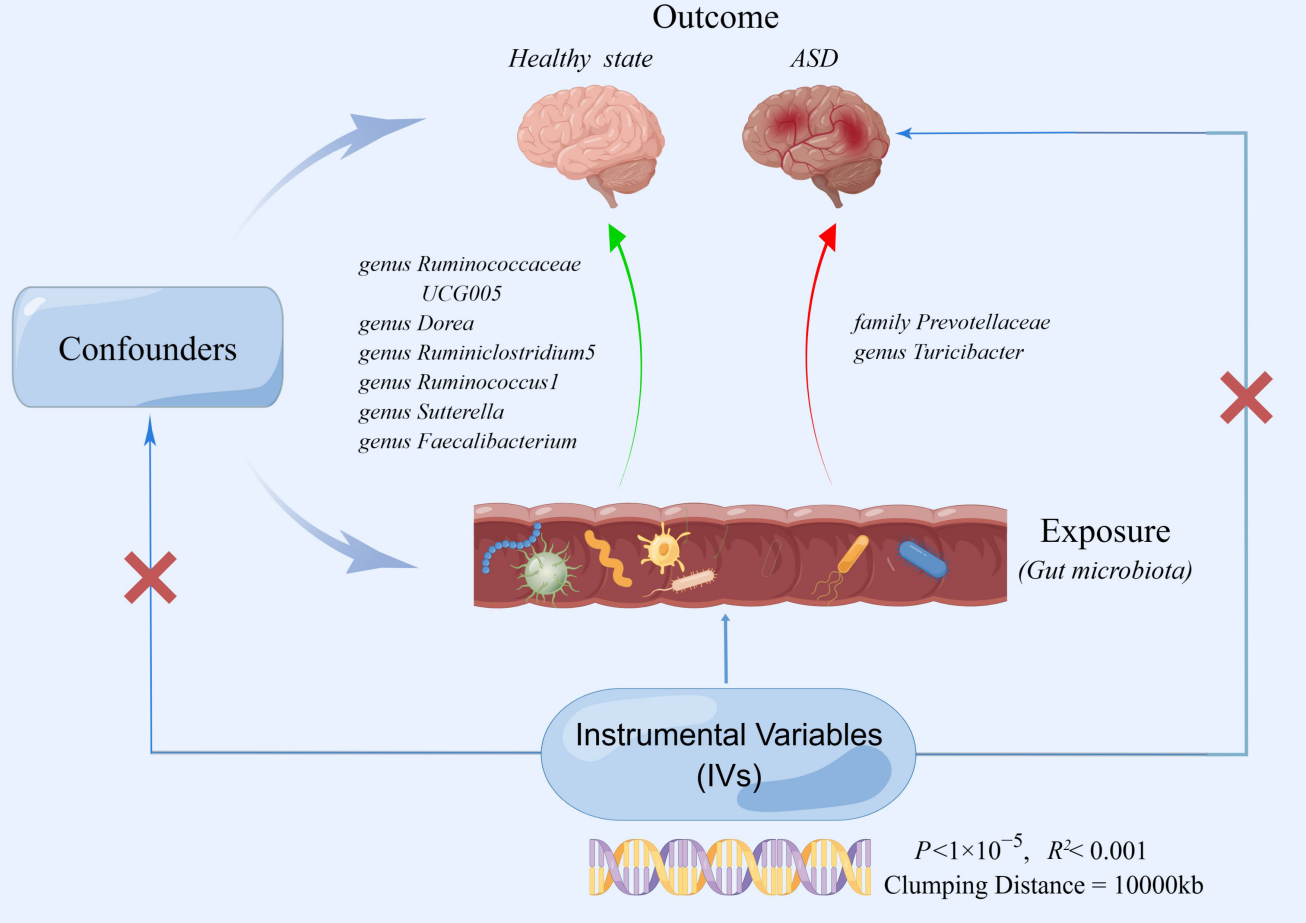
The two-sample MR study’s summary indicated the causal relationship between gut microbiota taxa and ASD. Five taxa of gut microbiota decreased the risk of ASD, whereas two taxa of gut microbiota accelerated the onset of ASD.

In this study, extensive MR analyses were carried out to determine the relationship between 206 gut microbiota taxa and ASD. **Figure 1** depicts the general design of the current investigation. Gut microbiota was the exposed component of interest (n=206), and the outcome was ASD. We then conducted a two-sample MR to determine if the gut microbiota and ASD are causally related by using IVW, simple mode, MR-Egger, weighted median, and weighted model. For MR studies, the following three assumptions should be satisfied: (I) the genetic variants used as instruments are strongly associated with the exposure of interest (the gut microbiota);(II) the genetic variants are not correlated with potential risk factors of the outcome (ASD) (*P*<1×10^-5^); (III) the genetic variants affect the outcome only through exposure (Emdin et al., 2017). In the present study, our analyses effectively addressed all of these hypotheses. We extracted robust instrument variables and estimated their strength by calculating their F statistics. As stipulated by Mendel’s second law, SNPs on chromosomes during meiosis were randomly assigned, the second assumption was satisfied by the study design (Veller et al., 2019). For the third assumption, we assessed the pleiotropy using the MR-Egger intercept and Mendelian Randomization Pleiotropy RESidual Sum and Outlier (MR-PRESSO) (Verbanck et al., 2018). We used publicly available summary statistics from previously published research. These summary statistics are free to download and can be used without limitations. Additionally, the original studies from which the summary statistics were derived had obtained proper ethical approval and informed consent. Consequently, no ethical sanction was required for this research.

### 2.2 Data sources for the exposure

In earlier research investigating the relationship between human genetic variants and gut microbiota, the MiBioGen consortium conducted a large-scale GWAS (Kurilshikov et al., 2021). This consortium integrated 16S gut microbiota data from 18,340 participants across 24 population-based cohorts representing ancestries from the USA, Canada, Israel, Germany, Sweden, Finland, and the UK (Kurilshikov et al., 2021). As exposure variables, we utilized data from the MiBioGen consortium’s 211 gut microbiota for this study. The GWAS detailed data of gut microbiotas may be downloaded from https://mibiogen.gcc.rug.nl/.

### 2.3 Data sources for the outcome

GWAS summary statistics for ASD were derived from the latest Psychiatric Genomics Consortium, of European ancestry (18,381 cases and 27,969 controls) (Grove et al., 2019). All patients with ASD were diagnosed based on the 10th version of the International Classification of Diseases (ICD 10) and received treatment at psychiatric hospitals or outpatient psychiatric clinics. This GWAS summary statistic of ASD may be downloaded from https://pgc.unc.edu/for-researchers/download-results/. After obtaining the SNPs’ information for exposure and outcome, we performed data harmonization to prepare the datasets for further analysis.

### 2.4 Selection of instrumental variables

As Instrumental Variables (IVs), SNPs strongly related with each gut microbiota were chosen. To obtain sufficiently robust results, we used *P* < 1×10^−5^ for gut microbiota identified by GWAS, as they rarely reached the significance level of the entire genome (*P* < 10^-8^) (Sanna et al., 2019). Besides, the MR method requires that there is no linkage disequilibrium (LD) between the selected IVs. To ensure no LD, we chose R^2^< 0.001 and Clumping Distance=10000 kb to preserve independent SNPs. Then, palindromic SNPs and SNPs that did not exist in outcomes were excluded. Finally, to avoid deviations induced by weak IVs, we calculated the F-statistic for every gut microbiota subsequent calculation methodology: F= R^2^× (n−k−1)/K× (1-R^2^) (Li et al., 2022; Xu et al., 2022). IVs with an F-value<10 was defined as weak and removed (Xie et al., 2023). For the functional MR analysis, we included only gut microbiota with three or more instrumental SNPs to ensure result stability, following the approach adopted in a previous study (Chen and He, 2023).

### 2.5 MR analysis

Five popular MR methods: IVW, MR-Egger, weighted median, simple mode, weighted model was used for the MR analysis (Burgess et al., 2013; Bowden et al., 2016; Burgess and Thompson, 2017; Verbanck et al., 2018). The IVW was the primary method to determine the relationships between the gut microbiota and ASD. The remaining MR analyses were utilized as supplementary statistical methods (Bowden et al., 2017). In order to acquire a precise interpretation of the causal link, The Benjamin Hochberg procedure was used to calculate the false discovery rate (FDR) to avoid false positive results, and the causal feature was defined at *P*_FDR_<0.10. Gut microbiota was deemed potentially causal if the IVW *P*<0.05 with MR analysis but lost significance after FDR correction. Taxa with a *P*_FDR_<0.10 were defined as solid causal associations. Furthermore, heterogeneity tests were carried out for statistically significant results using Cochran’s Q statistics. In the case of heterogeneity, we employed random-effect IVW models. If not, the IVW model with fixed effects was used (Shi et al., 2023). Meanwhile, we used MR-PRESSO and MR-Egger intercept tests to detect horizontal pleiotropy (Chen et al., 2023). Finally, the leave-one-out sensitivity analysis was performed to examine if one single SNP drove the causal association. R software (version 4.2.1) program is used for the above-mentioned statistical analysis. The “Two Sample MR package” was used for MR analysis, while the ‘MR-PRESSO’ package was utilized for the MR-PRESSO (Waters and Ley, 2019).

### 2.6 Reverse MR analysis

We also carried out a reverse MR analysis utilizing SNPs related to ASD as IVs to investigate if ASD has any causal influence on the major bacterial species identified in our study.

### 2.7 Enrichment analysis

We conducted enrichment analysis based on lead SNPs for the selected gut microbiota to comprehensively investigate the physiological effects of gut microbiota on ASD (Yu et al., 2021). We used the GWAS4D website (http://www.mulinlab.org/varnote/index.html) to map causative microbial taxonomic lead SNPs to nearby genes (Huang et al., 2018). Then, using the Metascape website (https://metascape.org), we conducted an enrichment analysis (Zhou et al., 2019). Functional enrichment analysis was performed using GO and Kyoto Encyclopedia of Genes and Genomes (KEGG) pathway analysis. Significantly enriched terms with *P*< 0.01, the least number >3, and an enrichment factor >1.5 were gathered and organized into clusters.

## 3 Result

### 3.1 Overview of Instrumental Variables for ASD

Five of the 211 gut bacteria had less than three SNPs. Due to the potential bias in sensitivity results when including such SNPs, these five gut bacteria were not included in our study (**Table S1**). After conducting quality control measures that involved screening of *P*<1×10^-5^, LD effects, palindromic, and calculating the F-statistic, a total of 206 gut microbiota were identified for further analysis. The IVW method has discovered eight gut microbiota taxa with relationships with ASD. However, the presence of pleiotropy was indicated by an Egger intercept *P*<0.05, which prevented us from establishing a causal connection between the *genus Faecalibacterium* and ASD (IVW *P* = 0.047). Finally, we identified seven gut microbiota and 83 SNPs associated with ASD.

### 3.2 Causative connection between gut microbiota and ASD

**Figure 2:**
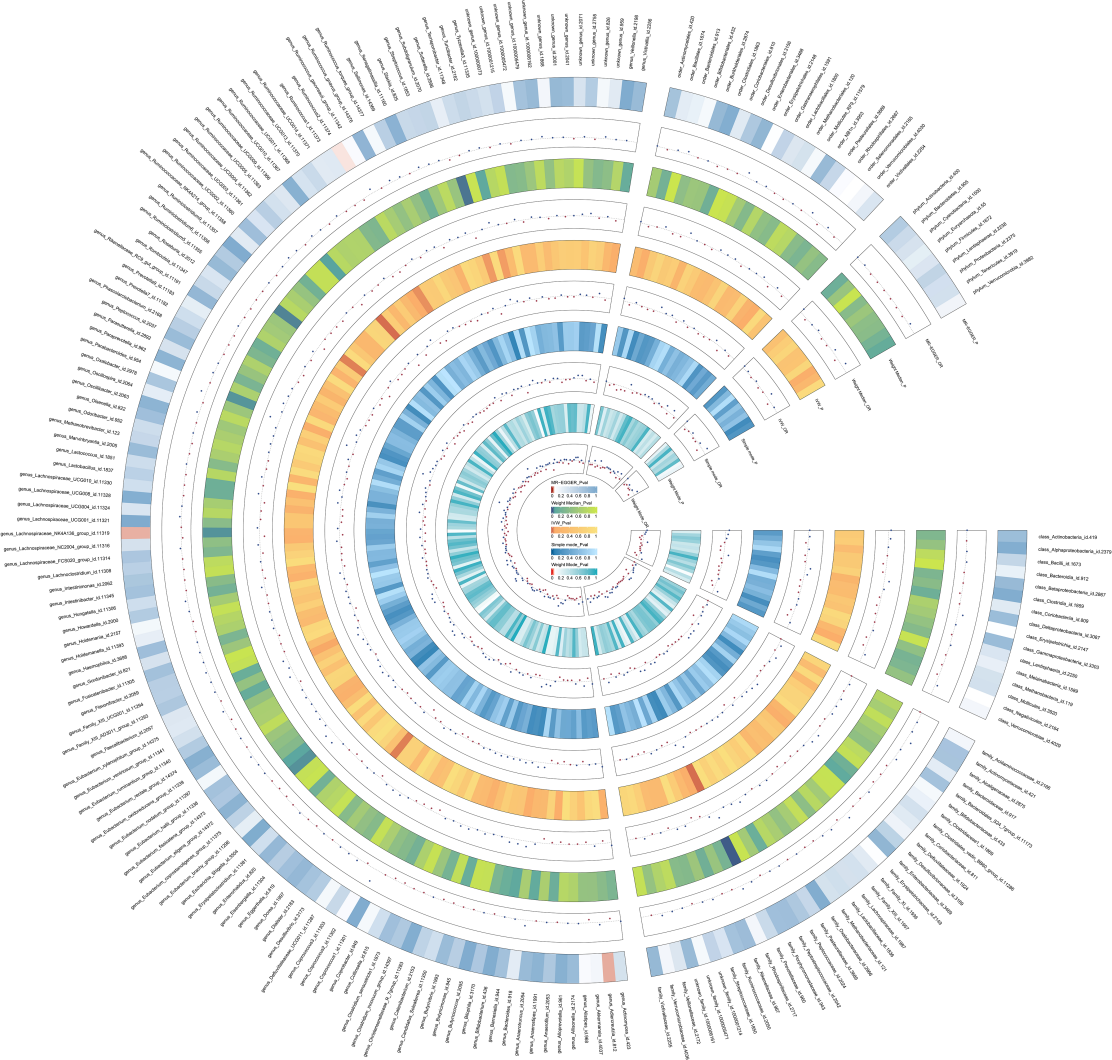
The detailed MR results for the associations between 206 bacterial taxa and ASD from the above five analysis methods. The estimations for the MR-Egger, Weight Median, IVW, Simple mode, and Weight Mode are shown as circles from outer to inner. Gut microbiota is classified in class, family, genus, order, and phylum. The color variations represented the size of the p-value. The scatter plots reflect OR, with OR> 1 labeled blue and OR < 1 labeled red.

The IVW method results revealed evidence of a significant connection between gut microbiota and ASD risk. Eventually, 7 gut microbiota were selected with significant level less than 0.05, including 1 family and 5 genera, as presented in **Table 1**. **Table 2** displays the other four additional MR analysis techniques results (MR-Egger, weighted median, simple model, and weighted model). However, after multiple-testing correction, strong relationships (*P_FDR_* < 0.10) were only observed between ASD and *family Prevotellaceae* (*P_FDR_* = 0.095), *genus RuminococcaceaeUCG005* (*P_FDR_* = 0.0953). The detailed results of 206 genetically gutted bacterial taxa from the above five analysis methods are illustrated in **Figure 2** and **Table S2**. Comprehensive data on all IVs are provided in **Table S3**. The results of instrumental SNPs for the causal gut microbiota’s impact on ASD are also provided in **Table S4**.

**Table 1:** IVW results between gut microbiota and ASD (*P*< 1×10^-5^).

Level	Gut Microbiota	Inverse variance weighted				
		nSNP	SE	*P*	OR	95% CI
genus	*Ruminococcaceae UCG005*	14	0.07	6.9×10^−4^	0.78	0.67-0.90
family	*Prevotellaceae*	15	0.06	9.2×10^−4^	1.24	1.09-1.40
genus	*Dorea*	10	0.09	1.4×10^−2^	0.81	0.69-0.96
genus	*Ruminiclostridium5*	11	0.09	1.5×10^−2^	0.81	0.69-0.96
genus	*Ruminococcus1*	10	0.08	2.8×10^−2^	0.83	0.70-0.98
genus	*Sutterella*	12	0.09	3.6×10^−2^	0.82	0.68-0.99
genus	*Turicibacter*	10	0.06	3.7×10^−2^	1.14	1.00-1.29

**Table 2:** The MR Egger, Weighted median, Simple mode and Weighted mode MR test estimates of the associations between Gut Microbiota and ASD.

Level	Gut Microbiota	MR Egger		Weighted median		Simple mode		Weighted mode	
		*P*	OR (95% CI)	*P*	OR (95% CI)	*P*	OR (95% CI)	*P*	OR (95% CI)
genus	*Ruminococcaceae UCG005*	0.38	0.818 (0.53-1.26)	0.05	0.83 (0.69-1.0)	0.24	0.83 (0.62-1.12)	0.18	0.83 (0.65-1.07)
family	*Prevotellaceae*	0.46	1.20 (0.75-1.90)	0.01	1.27 (1.07-1.51)	0.13	1.27 (0.95-1.69)	0.15	1.26 (0.94-1.70)
genus	*Dorea*	0.85	0.95 (0.60-1.52)	0.35	0.89 (0.71-1.13)	0.65	0.91 (0.62-1.33)	0.64	0.92 (0.67-1.27)
genus	*Ruminiclostridium5*	0.83	0.92 (0.46-1.85)	0.03	0.79 (0.64-0.98)	0.16	0.77 (0.55-1.08)	0.15	0.77 (0.56-1.07)
genus	*Ruminococcus1*	0.29	0.78 (0.50-1.21)	0.28	0.89 (0.73-1.10)	0.84	0.96 (0.68-1.36)	0.83	0.96 (0.68-1.37)
genus	*Sutterella*	0.24	0.55 (0.22-1.39)	0.02	0.77 (0.62-0.96)	0.09	0.66 (0.42-1.03)	0.09	0.67 (0.44-1.02)
genus	*Turicibacter*	0.52	1.20 (1.71-2.03)	0.07	1.16 (0.99-1.38)	0.25	1.19 (0.90-1.57)	0.27	1.18 (0.89-1.57)

**Figure 3:**
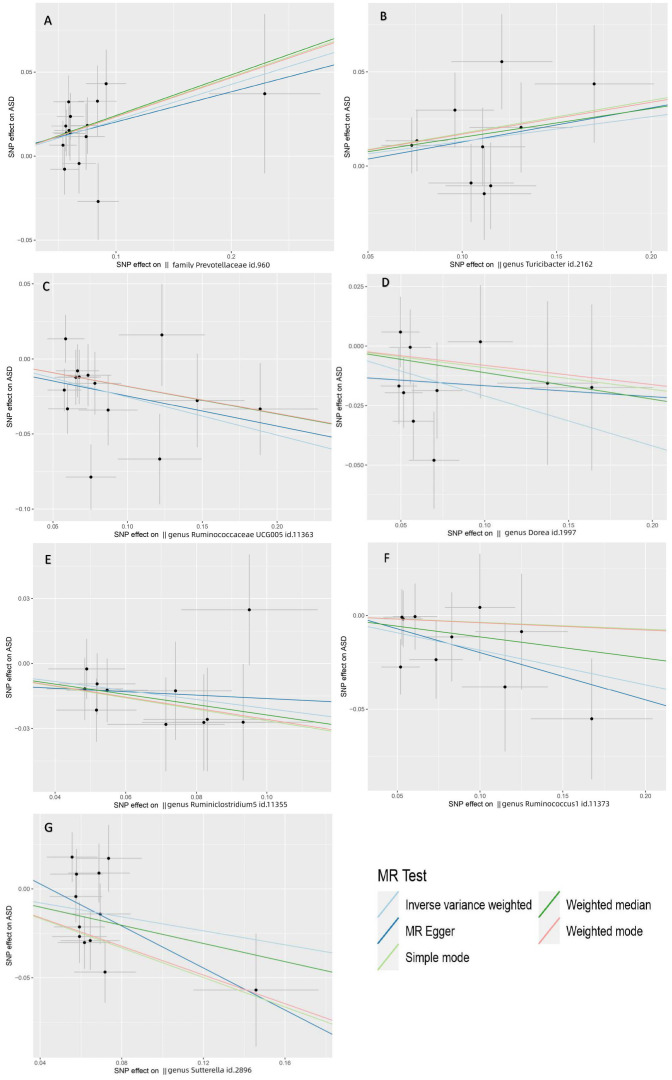
Scatter plots depicting the MR estimations for the substantial causal effects of 7 gut microbiota on the risk of ASD. The Gut microbiota from **(A-G)** are The Gut microbiota from **(A-G)** are *family Prevotellaceae, genus Turicibacter, genus Ruminococcaceae UCG005, genus Dorea, genus Ruminiclostridium5, genus Ruminococcus1 and genus Sutterella*. Positive correlation lines travel diagonally upward from left to right, demonstrating that the gut microbiome facilitates ASD. Negative correlation lines travel diagonally downhill from left to right, demonstrating that gut bacteria can lower the incidence of ASD. The horizontal and vertical lines represent the 95% confidence intervals for each association.

Based on the results of IVW testing and FDR multiple-testing correction, we found that the *family Prevotellaceae* (IVW OR=1.24; 95% CI: 1.09-1.40, *P*=9.2×10^-4^) was strongly positively correlated with ASD (*P_FDR_* = 0.095), implying that *Prevotellaceae* serve an important role in increasing the occurrence of ASD (**Figure 3A**). Likewise, the Weighted median yielded identical results. The IVW results also suggested a possible positive association between the *genus Turicibacter* and ASD (IVW OR=1.14, 95% CI: 1.00-1.29, *P* = 3.7×10^−2^) (**Figure 3B**). Nonetheless, the *P*-values of the *genus Turicibacter* for WM and MR-Egger were not statistically significant.

The *genus RuminococcaceaeUCG005* (IVW OR=0.78, 95% CI: 0.67-0.89, *P*=6.9×10^−4^) was strongly negatively correlated with ASD (*P_FDR_* = 0.0953), which suggested that *RuminococcaceaeUCG005* could reduce the risk of ASD (**Figure 3C**). In the meantime, we found four gut microbiota with a potential protective effect on ASD: *genus Dorea* (OR=0.81, 95% CI: 0.69-0.96, *P*=1.4×10^−2^), *genus Ruminiclostridium5* (OR=0.81,95% CI:0.69-0.96, *P*=1.5×10^−2^), *genus Ruminococcus1* (OR=0.83, 95% CI: 0.70-0.98, *P*=2.8×10^−2^), and *genus Sutterella* (OR=0.82, 95% CI: 0.68-0.99, *P*=3.6×10^−2^) (**Figures 3D-G**).

### 3.3 Horizontal pleiotropy and heterogeneity testing

We used MR-PRESSO and MR-Egger intercept to assess horizontal pleiotropy effects and Cochran’s Q statistics for heterogeneity testing. A *P*-value >0.05 indicated no heterogeneity or pleiotropy. Sensitivity analysis of *genus Ruminococcaceae*, *family Prevotellaceae*, *genus Dorea*, *genus Ruminiclostridium5*, *genus Ruminococcus1*, *genus Sutterella*, and *genus Turicibacter* using MR-Egger intercept and MR-PRESSO showed that none of the above flora had horizontal pleiotropy (*P*<0.05). Similar to the horizontal pleiotropy results, Cochran’s Q test revealed no heterogeneity (*P*<0.05). Therefore, fixed-effect IVW model was applied in this study (**Table S5**). Finally, we performed the leave-one-out analysis by removing each instrumental SNP to ensure that no single SNP heavily influenced the causal estimates. Forest and funnel plots were generated for causal and suggestive gut microbiota (**Figure S1, 2**). No SNP had a substantial effect size on the study’s estimation, indicating the robustness of the findings (**Figure S3**). The above findings demonstrate a consistent, genetically-based causal relationship between the gut microbiome and ASD.

### 3.4 Reverse MR analysis

Instrumental SNPs (*P*<5×10^-6^, R^2 =^ 0.001, Clumping Distance = 10000 kb) were chosen for ASD phenotypes. The MR analysis was done to determine the reverse causal correlation of ASD with the identified gut microbiota. According to the results of reverse MR analysis, there was no discernible link between ASD and the gut bacteria (**Table S6**). Cochran’s Q test showed no heterogeneity. Besides, the MR-Egger intercept analysis results in no horizontal pleiotropy (**Table S7**).

### 3.5 Enrichment analysis

**Figure 4:**
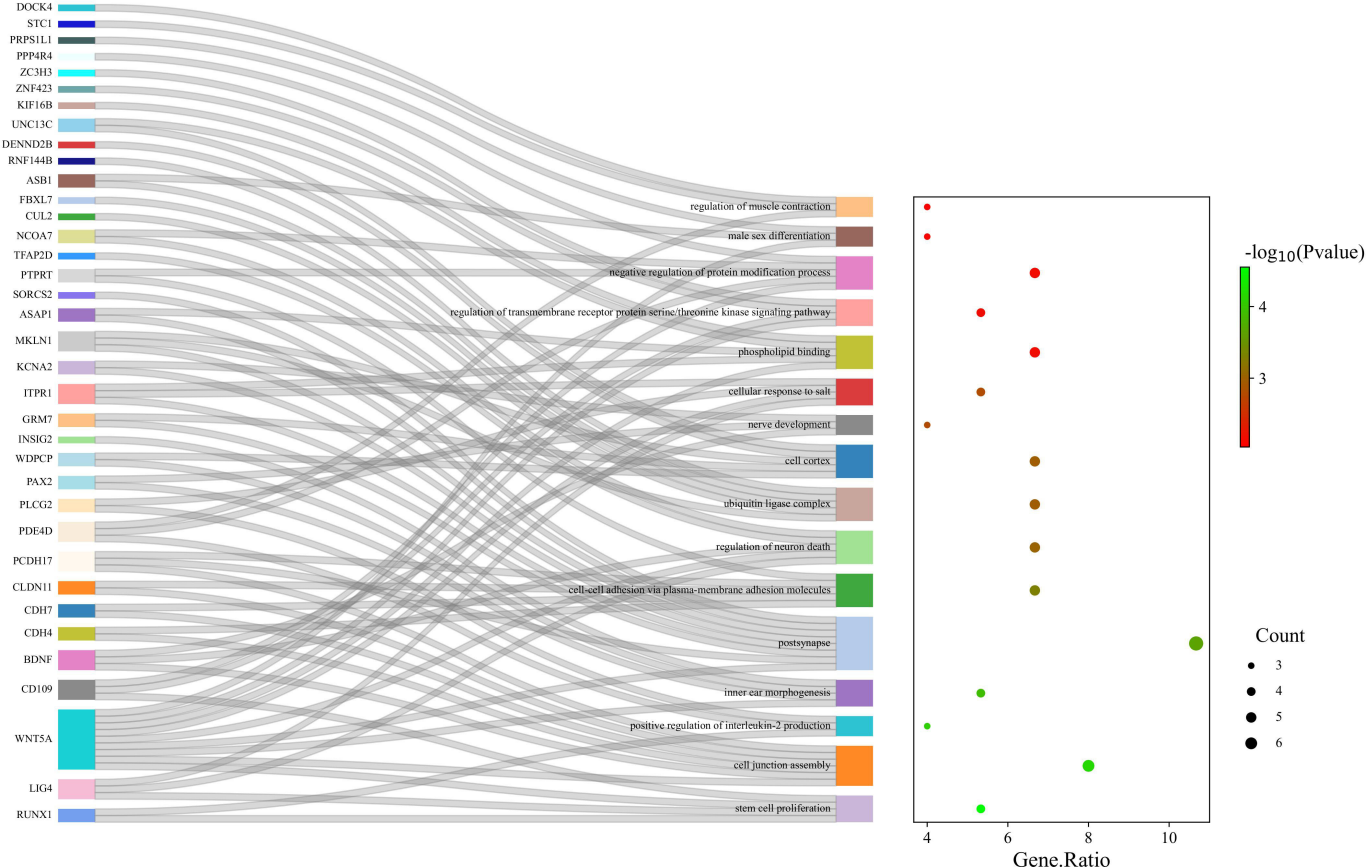
GO enrichment analysis of the gut microbiota of ASD; 16 pathways were significantly enriched.

Enrichment analysis of gut microbiota on ASD yielded 109 regulatory pathways. 16 significantly enriched pathways were selected. (**Table S8**, **Figure 4**, **Figure S4**)

## 4 Discussion

In our research, we have discovered through conducting a bidirectional two-sample MR analysis that specific gut microbiota had correlation with ASD. Using IVW, MR Egger, simple mode, weighted median, and weighted model analysis methods, we successfully identified that the *family Prevotellaceae*, *genus Turicibacter* had a potential positive link with ASD, while *genus RuminococcaceaeUCG005*, *Ruminiclostridium5*, *Ruminococcus1*, and *Sutterella* had potential protective effects against ASD. After FDR multiple-testing correction we further observed that the *family Prevotellaceae* exhibited a significant positive correlation with ASD, while the *genus RuminococcaceaeUCG005* displayed a strong negative correlation. Overall, our findings pinpoint specific gut microbiota that had correlation with ASD, which elucidates the potential biological processes of gut microbiota in the development of ASD from a genetic perspective by using GO analysis. This finding may help lead to microbiological interventions in future ASD clinical trials.

According to the recent study, autistic children have dissimilar gut microbiome than normal developing children (Liu et al., 2019a). The gut microbiota, the “second human genome,” might play a key role in the development of ASD by significantly influencing the gut microbiota-brain axis (Grice and Segre, 2012, Yu and Zhao, 2021). The present studies have determined the involvement of chemical, metabolic, and endocrine pathways in the pathogenesis of ASD (Sharon et al., 2019; Needham et al., 2021). Notably, individuals with ASD exhibit signs of low-grade inflammation, which suggests that gut microbiota may have some effect on the immune system and consequently induces neuroimmune responses in the central nervous system (Kim et al., 2017). The significant influence of microbial metabolites including bile acid derivatives and short-chain fatty acids (SCFAs), on patient’s symptoms with ASD cannot be overlooked (Morais et al., 2021). Innovative therapeutic approaches, encompassing diet therapy, probiotic therapy, fecal microbiota transplantation, and targeted antimicrobial therapy, might serve as promising strategies to alleviate the linguistic, cognitive, and behavioral deficiencies associated with ASD (Kang et al., 2017; Karhu et al., 2020). Thus, investigating the pathogenesis of ASD from the perspective of gut microbiota remains crucial.

Our research demonstrates a substantial correlation between the presence of the *family Prevotellaceae* and the incidence of ASD. Research indicates that the *family Prevotellaceae* can promote inflammation, with its presence in the gut being associated with autoimmune diseases and chronic intestinal inflammation (Zhou et al., 2020; Kim et al., 2021). Upon colonization in the gastrointestinal tract, these bacteria infiltrate the mucosal layer of the large intestine, and eventually settle in the colonic crypts (Elinav et al., 2011). This penetration into ordinarily sterile areas can escalate the activation of the innate immune system, trigger specific T-cell and antibody responses, and consequently foster chronic intestinal inflammation or exacerbate existing colitis (Elinav et al., 2011; Scher et al., 2013; Palm et al., 2014). Additionally, the activation of a pro-inflammatory environment can lead to neuroinflammation, potentially impacting the development and differentiation of microglial cells within the nervous system (Morais et al., 2021). Given the prevalent mild inflammatory responses observed in children with ASD (Morais et al., 2021), it is hypothesized that the impact of the *family Prevotellaceae* on the immune system may influence neuroimmune interactions in ASD patients, subsequently leading to neurological symptoms.

*Genus Turicibacte*r is a critical component of the gut microbiota, and recent research suggests its potential role in influencing intestinal health (Zhong et al., 2015). Enhanced serotonin signaling has been observed to reverse detrimental symptoms in various ASD mouse models (Walsh et al., 2021). However, *genus Turicibacter* fermentation enhances the inhibitory effects of serotonin signaling proteins, Wnt signaling proteins, and the expression of downstream genes (Lin et al., 2023). These findings corroborate our hypothesis that *genus Turicibacter* might adversely affect ASD through chemical, metabolic, and endocrine pathways.

Beyond the aforementioned gut microbiota associated with ASD, our investigation has pinpointed five gut microbiota that exhibit protective properties: *genus RuminococcaceaeUCG005, genus Ruminiclostridium5, genus Dorea, genus Sutterella*, and *genus Ruminococcus1*. Our results indicate a significant negative correlation between *RuminococcaceaeUCG005* and ASD. This correlation is noteworthy since this bacterium can produce the SCFAs, butyrate, and is widely considered beneficial for gut health (Min et al., 2023). Literature indicates a reduced concentration of butyrate in children diagnosed with ASD (Liu et al., 2019b). Butyrate possesses neuroprotective qualities, modulating the production of catecholamines and neurotransmitters within the nervous system (De Angelis et al., 2015). Additionally, it promotes memory consolidation through epigenetic mechanisms and maintains neural plasticity (Mohajeri et al., 2018). Research has demonstrated that a sustained low-butyrate environment can diminish PPARα expression in colonic macrophages, consequently inhibiting NF-κB signaling and promoting the release of pro-inflammatory cytokines IL-1β and TNF-α (Chen et al., 2022). Thus, we hypothesize that the protective effect of *genus RuminococcaceaeUCG005* against ASD is mediated through a synergistic interaction of microbial metabolites and neuroimmune responses.

Moreover, existing literature suggests that *genus Dorea* may contribute to mitigating tropomyosin (Tm)-induced allergic reactions (Fu et al., 2017). *Genus Dorea* possesses the potential to modulate mucosal metabolism, thereby affecting intestinal permeability. A negative correlation has been observed between *genus Dorea* and food allergies in infants (Savage et al., 2018; Zhou et al., 2021). Given the increased incidence of food allergies in children with ASD, we hypothesize that the protective effect of *genus Dorea* against ASD may be related to its capacity to alleviate Tm-induced allergic responses (Schieve et al., 2012; Xu et al., 2018). Research indicates that the abundance of *genus Sutterella* is higher in individuals without any diseases compared to those with Crohn’s disease (Gevers et al., 2014). Cohort studies have shown that aggregation of Suterella is inversely proportional to the host inflammatory response (Morgan et al., 2015). Consequently, it is plausible that the protective role of *genus Sutterella* against ASD may be achieved through reducing intestinal inflammation and modulating neuroimmune responses.

**Figure 5:**
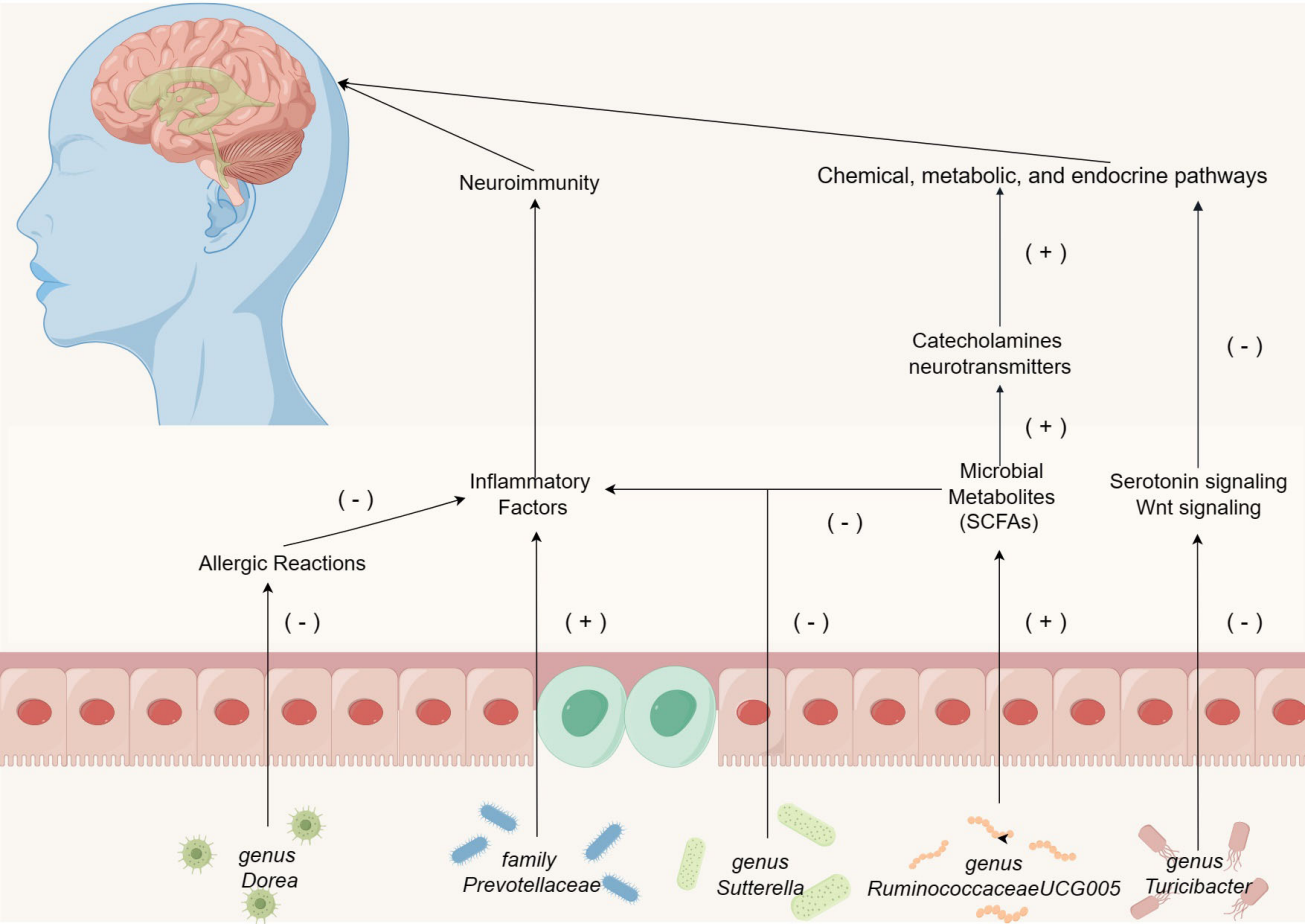
The mechanism of action of the microbiota (positive and negative) in ASD identified by the MR method, except for *genus Ruminiclostridium5* and *genus Ruminococcus1*.

In our study, we identified previously unreported gut bacteria, *genus Ruminiclostridium5* and *genus Ruminococcus1*, associated with ASD. In addition to the above two newly discovered gut microbiota, the mechanism of action of the microbiota (positive and negative) in ASD identified by the MR method has been briefly illustrated in **Figure 5**. The pathophysiological roles of these bacteria in ASD patients warrant further in-depth investigation. While current observational studies have not yet established a connection between these bacteria and ASD, our findings provide essential guidance for subsequent research. Furthermore, the potential effects of the aforementioned gut microbiota and their metabolites on the nervous system remain to be elucidated, necessitating additional observational studies for clarification.

Additionally, enrichment analysis demonstrated that several GO-related biological processes were significantly involved in the association between gut microbiota and ASD. The absence of certain microorganisms has been linked to enduring effects on neurogenesis, synaptogenesis, axonal and dendritic growth, and the establishment of neural connections, all of which can influence the development of ASD (Rogers et al., 2016). Research indicates that infants at higher risk of ASD suffer different gut microbiota composition during a crucial developmental period for the gut microbiota and brain (Zuffa et al., 2023). This indicates that alterations in gut microbiota during early development may have persistent impacts on the formation of neuronal pathways. Recent research highlights that a more diverse gut microbiome in infants at birth can foster stem cell proliferation through microbial metabolites, influencing infant development (Socha-Banasiak et al., 2021). This is consistent with the findings of our gene GO analysis. Besides, abnormalities of key genes in the cell junction assembly pathway can cause the development of ASD (Zhang et al., 2022). Through the action of bile acids and SCFAs, gut microbiota can also alter the energy metabolism of intestinal epithelial mitochondria, activate immune system cells, and modulate intestinal epithelial barrier function (Dunislawska et al., 2023). Changes in early-life gut microbiota can impact host neuroimmunoregulation (Morais et al., 2021). It is now understood that ASD patients typically exhibit a dysregulated intestinal epithelial barrier (Gonzales et al., 2021). Similarly, an observational study found that SCFAs produced by gut microbiota metabolism could modulate the production of interleukin-2 (IL-2), and autistic patients exhibited an abnormally high level of IL-2, consistent with our GO analysis results (Vinolo et al., 2011; Cao et al., 2021). Therefore, we hypothesize that intestinal microorganisms may cause ASD via their influence on early neurodevelopment, metabolic and endocrine pathways and induction of intestinal inflammation mediated by SCFAs.

Currently, employing mathematical algorithms to investigate the origins of diseases is a prevalent approach (Morton et al., 2023). Several studies have made notable advancements in etiological exploration using MR methods (Li et al., 2023; Luo et al., 2023; Sha et al., 2023; Yuan et al., 2023). MR offers an optimal research design, utilizing genetic variants as instrumental variables to elucidate causal relationships between potential risk factors (exposures) and diseases of interest (outcomes), effectively circumventing biases caused by confounding factors (Burgess and Labrecque, 2018). Our research introduces several innovations: 1. By leveraging bidirectional MR, we bypass common biases inherent in observational experiments and, from a genetic perspective, identify specific gut microbes that correlation with ASD. 2. Our study identifies new gut bacteria related to ASD that have not been recognized in observational research, providing a reference for further investigation into the interplay between gut microbiota and ASD. 3. Through GO analysis, we indirectly infer the biological processes through which gut microbiota influences ASD, shedding light on the potential physiological mechanisms underlying ASD from another perspective.

However, our study does have inherent limitations. First, the GWAS data related to gut microbiota were derived from a diverse cohort of 18,340 participants from multiple ethnic backgrounds, while the GWAS summary statistics for ASD were solely from individuals of European descent. Nevertheless, given that nearly 80% of the gut microbiota GWAS data originate from European populations, and that the dataset is extensive, diverse, and broadly representative, we proceeded to use this GWAS data for our research. Moreover, this dataset has been widely employed in existing MR studies. Lastly, due to insufficient data, we were unable to conduct detailed stratified analyses, such as those based on age and gender. As a result, we could not investigate potential disparities across different populations. Future research will need to address these limitations and shortcomings.

## 5 Conclusion

In this work, by leveraging bidirectional MR, we bypass common biases inherent in observational experiments and, from a genetic perspective, identify specific gut microbes that correlation with ASD. we successfully identified that the *family Prevotellaceae* was shown to be substantially positively connected with ASD, whereas the *genus RuminococcaceaeUCG005* was found to be strongly negatively correlated with ASD. The *genera Dorea, Ruminiclostridium5, Ruminococcus1*, *Sutterella* and *Turicibacter* have potential effect on ASD. Our study opens up new avenues for therapeutic interventions in this patient population. However, we need to do more study to explore the specific physiological mechanisms and significance of individual bacterial taxa in ASD pathophysiology.

## Data availability statement

The original contributions presented in the study are included in the article/**Supplementary Material**. Further inquiries can be directed to the corresponding author.

## Ethics statement

All participating studies involved in the GWAS obtained informed consent from the study populations. As we utilized publicly available datasets to conduct MR, no additional ethics approval was required.

## Author contributions

ZL: Data curation, Formal Analysis, Project administration, Software, Writing – original draft. SL: Data curation, Formal Analysis, Supervision, Validation, Writing – review & editing. FL: Supervision, Validation, Writing – review & editing. ND: Data curation, Project administration, Writing – original draft. RL: Data curation, Writing – original draft. SL: Data curation, Writing – original draft. LB: Data curation, Writing – original draft.
