# Supplementary material for: Gut microbiota and autism spectrum disorders: a bidirectional Mendelian randomization study
Source: Front Cell Infect Microbiol. 2023 Dec 14;13:1267721. doi: 10.3389/fcimb.2023.1267721 (PMC10753022; doi:10.3389/fcimb.2023.1267721)
Supplement: Supplementary file 1 [file DataSheet_1.docx]

Supplemental Files

Supplemental Tables

**Table S1.** The gut microbial with SNPs less than 3.

**Table S2.** MR analysis of all gut microbiota identified in MR analysis

**Table S3.** Instrument variables for gut microbiota taxa enrolled in this Mendelian randomization study

**Table S4.** List of instrumental SNPs of the causal gut microbiota taxa on ASD and their nearest genes

**Table S5.** The results of the MR analysis that indicate significant gut microbiota

**Table S6.** Reverse MR results of gut microbiota-ASD associations

**Table S7.** Horizontal pleiotropy and Heterogeneity testing result of Reverse MR analysis

**Table S8**. Significantly enriched pathways

Supplemental Figures


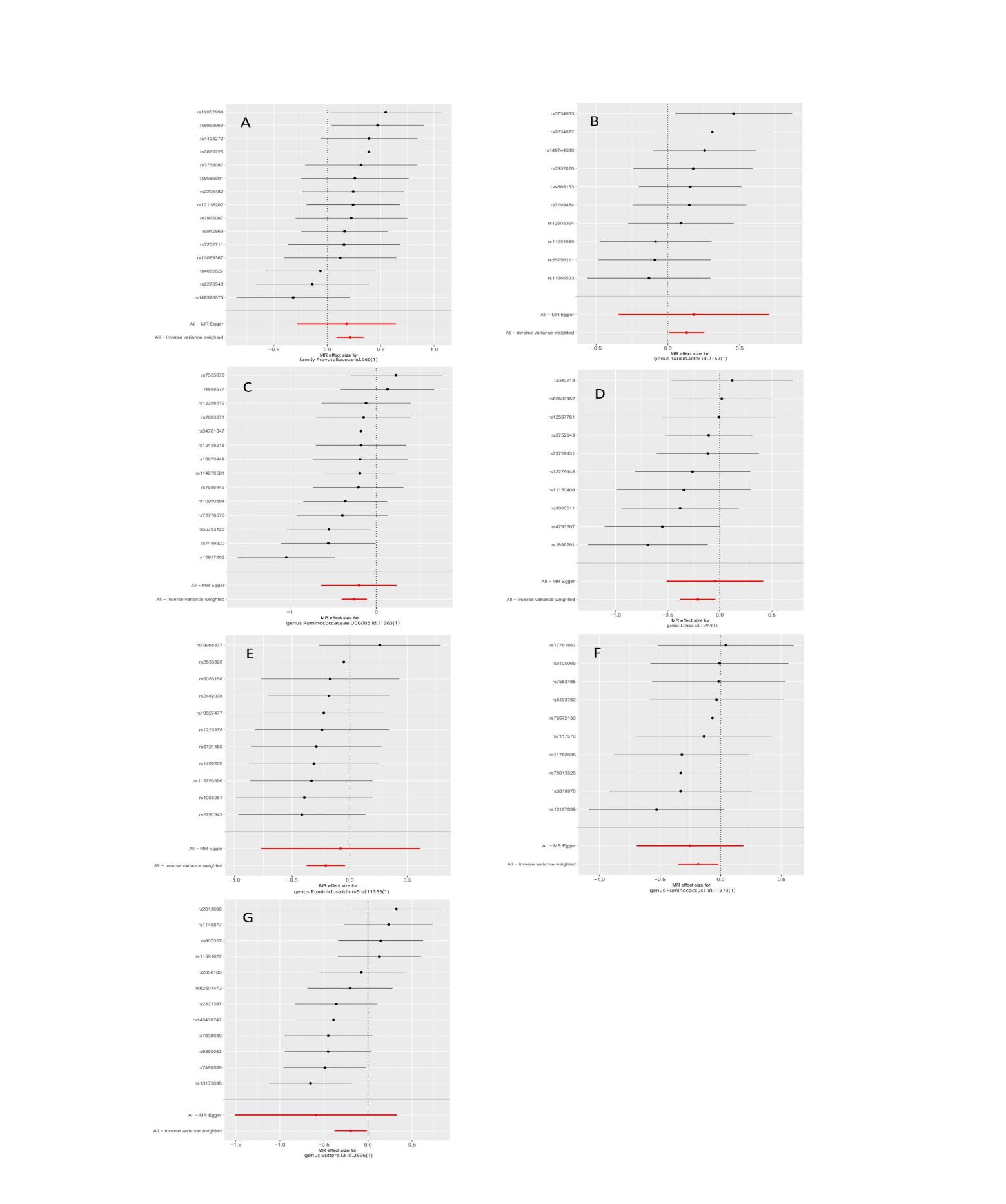


Figure S1. Forest plot of Mendelian randomization estimates between Gut microbiota and ASD.(A) Forest plot for effect of *family Prevotellaceae*; (B) Forest plot for causal effect of *genus Turicibacter;* (C) Forest plot for causal effect of *genus Ruminococcaceae UCG005* on ASD; (D) Forest plot for causal effect of *genus Dorea* on ASD; (E) Forest plot for causal effect of *genus Ruminiclostridium5* on ASD; (F) Forest plot for causal effect of *genus Ruminococcus1* on ASD; (G) Forest plot for causal effect of *genus Sutterella* on ASD;


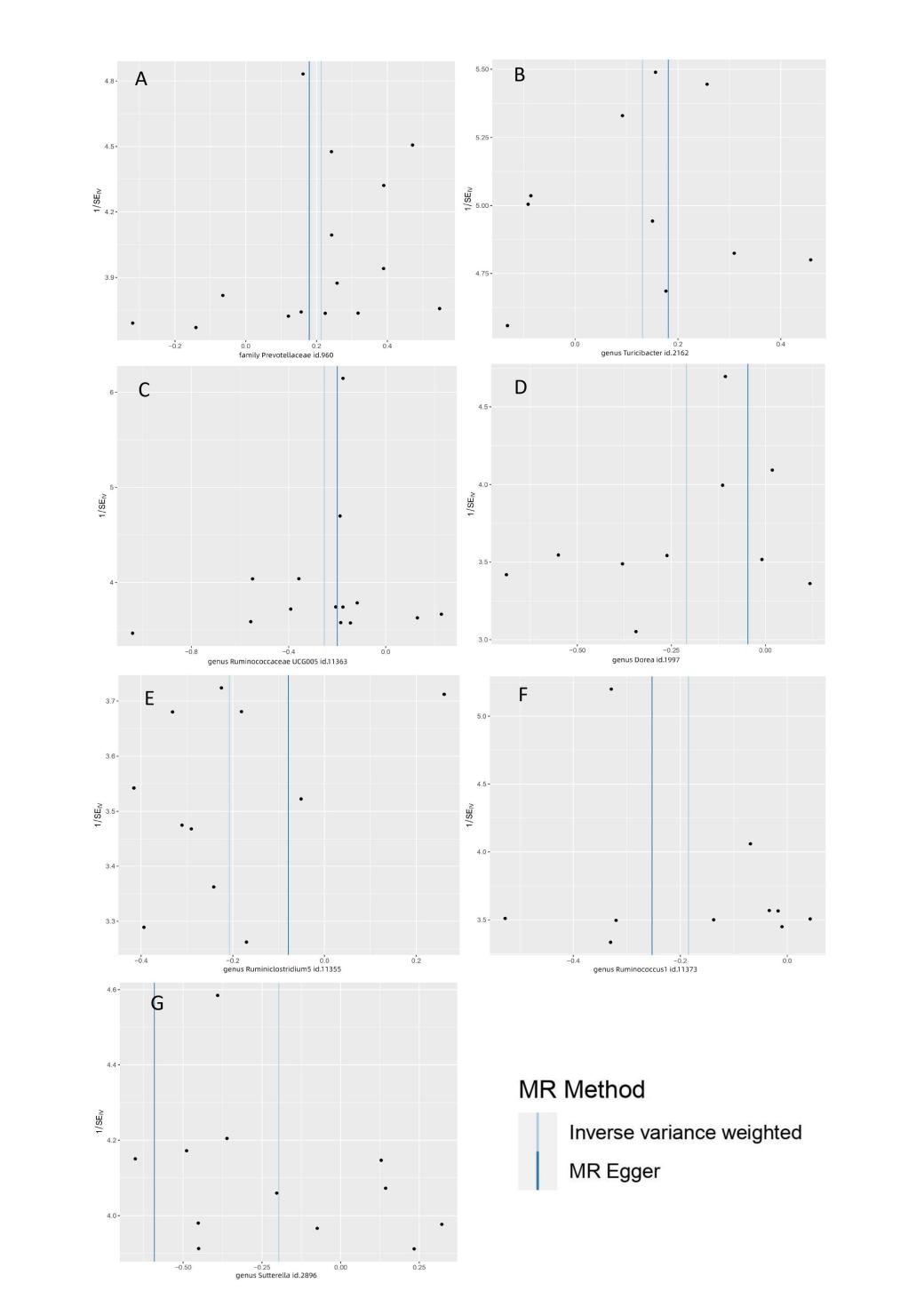


Figure S2. Funnel plots plot of Mendelian randomization estimates between Gut microbiota and ASD.(A) Funnel plot for effect of *family Prevotellaceae*; (B) Funnel plot for causal effect of *genus Turicibacter;* (C) Funnel plot for causal effect of *genus Ruminococcaceae UCG005* on ASD; (D) Funnel plot for causal effect of *genus Dorea* on ASD; (E) Funnel plot for causal effect of *genus Ruminiclostridium5* on ASD; (F) Funnel plot for causal effect of *genus Ruminococcus1* on ASD; (G)Funnel plot for causal effect of *genus Sutterella* on ASD;


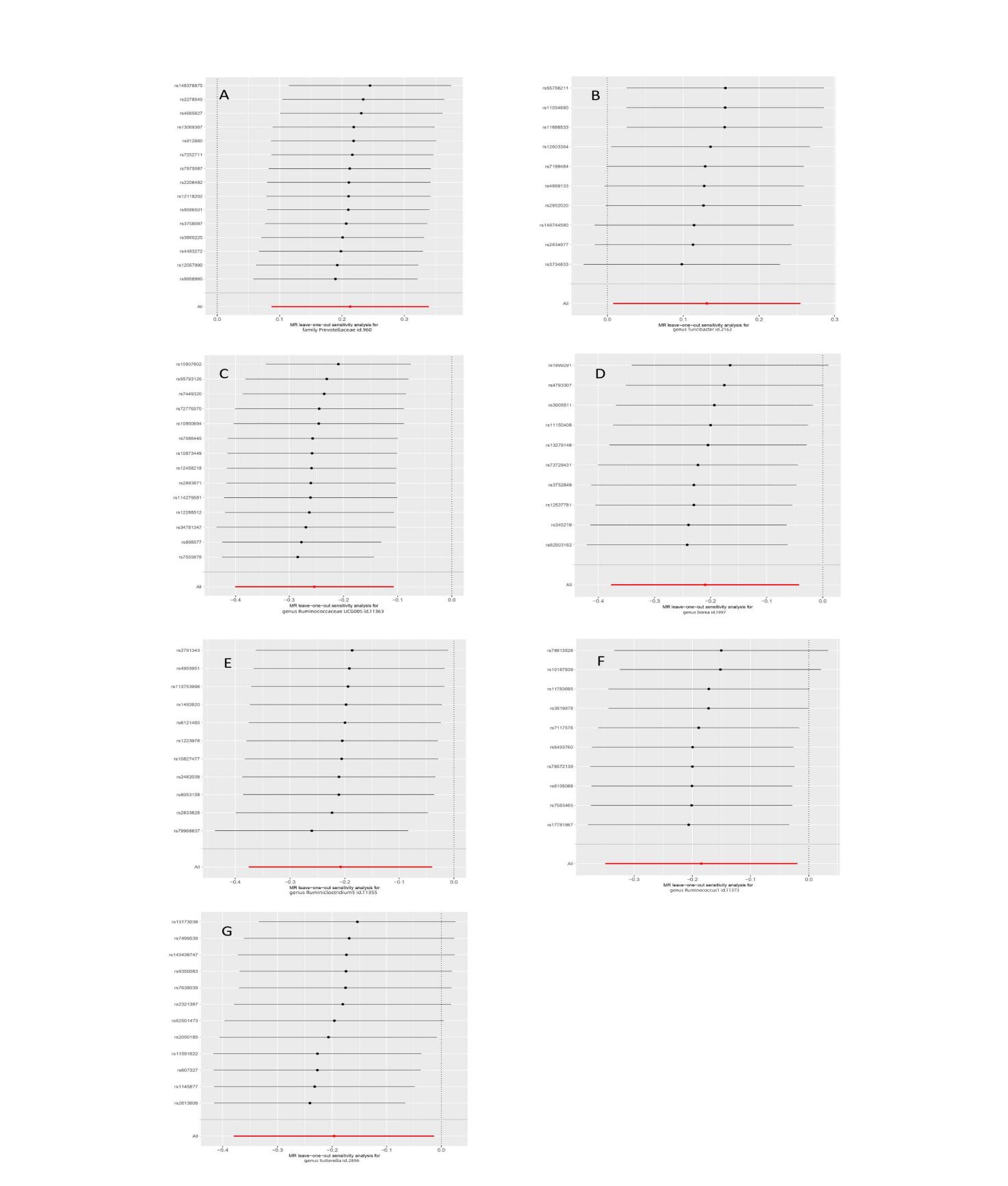


Figure S3. Leave-one-out result of Mendelian randomization estimates for the estimates of 7 gut microbiota taxa on the risk of ASD. Forest plot illustrating the effect estimate of the remaining SNPs after removing each SNP.The Gut microbiota from A to G are *family Prevotellaceae, genus Turicibacter, genus Ruminococcaceae UCG005, genus Dorea, genus Ruminiclostridium5, genus Ruminococcus1, genus Sutterella.*


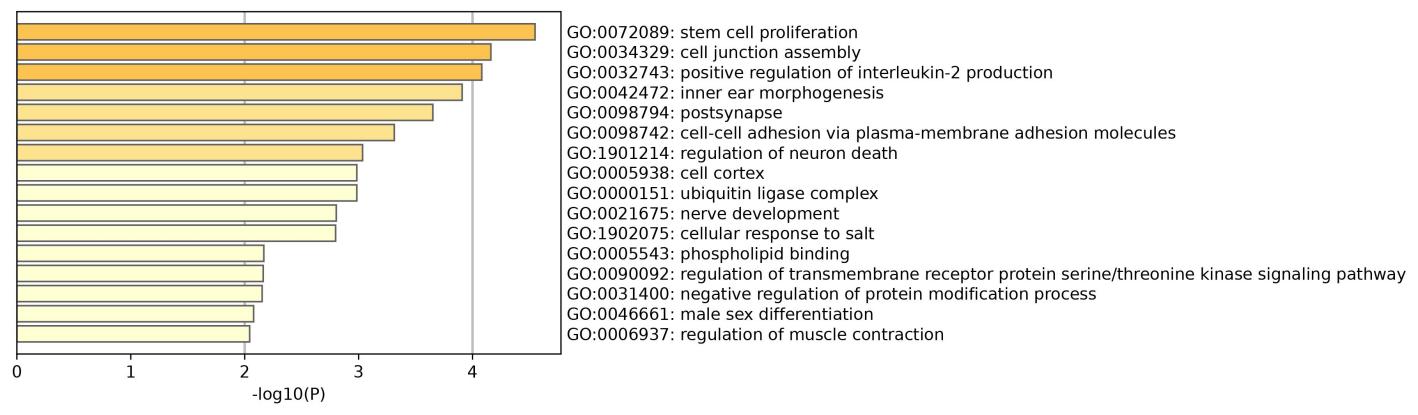


[**Figure**](https://www.ncbi.nlm.nih.gov/pmc/articles/PMC10248501/figure/f1/) **S4**. GO enrichment analysis of the 7 gut microbiota taxa on the risk of ASD, a total of 16 pathways were found to be significantly enriched.
